# Peripheral Blood Inflammatory Markers Can Predict Benign and Malignant Thyroid Nodules

**DOI:** 10.1155/2022/2319660

**Published:** 2022-06-26

**Authors:** Yuanyuan Deng, Jie Zhang, Guilin Zou, Shanshan Li, Zhaoxia Gong, Guanru Yue, Ping Fan, Jixiong Xu

**Affiliations:** ^1^Department of Endocrinology and Metabolism, First Affiliated Hospital of Nanchang University, Jiangxi Clinical Research Center for Endocrine and Metabolic Disease, Nanchang 330006, China; ^2^Department of Basic Medicine, Jiangxi Medical College, Nanchang University, Nanchang 330006, China

## Abstract

**Objective:**

Inflammation is related to the occurrence and development of various cancers. This study was designed to explore the role of peripheral blood platelet count, neutrophil-lymphocyte ratio (NLR), platelet count-lymphocyte count ratio (PLR), systemic inflammation index (SII), and other inflammatory markers in predicting benign and malignant Thyroid Imaging Reporting and Data System (TI-RADS) grade 3 thyroid nodules.

**Methods:**

In this retrospective study, 514 patients with TI-RADS grade 3 thyroid nodules were enrolled. According to the pathological results, the patients were divided into the benign and malignant nodule groups. We compared the clinical characteristics between the two groups and analysed the influencing factors for malignant thyroid nodules by univariate and stepwise multivariate logistic regression analyses and then analysed the cutoff value of each influencing factor according to the receiver operating characteristic curve.

**Results:**

The leukocyte count, neutrophil count, platelet count, NLR, PLR, and SII of the malignant nodule group were significantly higher than those of the benign nodule group (*P* < 0.05), the age and the diameter of nodule of the malignant nodule group were significantly smaller than those of the benign nodule group (*P* < 0.05). After excluding the influence of confounding factors, SII (odds ratio (OR) = 1.006; 95% confidence interval (CI) = 1.003–1.008; *P* < 0.001), PLR (odds ratio (OR) = 0.981; 95% confidence interval (CI) = 0.981–0.992; *P* < 0.05), leukocyte count (odds ratio (OR) = 0.654; 95% confidence interval (CI) = 0.466–0.892; *P* < 0.05), and age (OR = 0.969; 95% CI = 0.954–0.985; *P* < 0.001) were independent risk factors for malignant thyroid nodules, and the cutoff value of SII and PLR in predicting benign and malignant thyroid nodules were 545.63 × 10^9^/L and 138.63.

**Conclusion:**

This study showed that peripheral blood SII, PLR, leukocyte count and age were independent risk factors for malignant thyroid nodules, and the combination of these can better predict benign and malignant thyroid nodules, which can further guide the diagnosis and treatment of TI-RADS grade 3 thyroid nodules.

## 1. Introduction

Thyroid nodules are a common clinical disease caused by various factors [1]. Recently, with the improvement in living standards, people's attention to health, the increase in physical examination rates, obesity, environmental exposure, and other factors have caused a significant increase in its incidence [2, 3]. Consistent with the trend in the incidence of thyroid nodules, thyroid cancer incidence and deaths due to thyroid cancer are also on the rise [4, 5]. Thyroid function examinations, ultrasonography, and fine-needle aspiration are the most commonly used examination methods for clinically assessing benign and malignant thyroid nodules, but there is still a certain rate of missed diagnosis [6]. Therefore, recently, thyroid contrast-enhanced ultrasonography, thick-needle biopsy, molecular testing, genetic testing, and other tests have been added to the cohort to distinguish benign thyroid nodules from malignant ones. Although these methods can increase the diagnostic rate to some extent, some inspection items are expensive, and some are invasive, which will cause certain psychological and economic pressure on individuals, and there is the suspicion of excessive use of inspections [7–9]. Presently, it is an art that endocrinologists and thyroid surgeons should master to balance the relationship between benign and malignant thyroid nodules and overexamination.

Ultrasonography has become the preferred method for inspecting thyroid nodules. The Thyroid Imaging Reporting and Data System (TI-RADS) classifies thyroid nodules according to their characteristics to predict the benignancy and malignancy of these thyroid nodules [1, 10]. Presently, TI-RADS grade 3 thyroid nodules are considered benign lesions and do not require excessive diagnosis and treatment. However, there will also be a small probability (<5%) of malignant nodules among benign lesions [11], and the discovery of thyroid nodules will bring a certain amount of pressure to the patient. Therefore, simple and further diagnosis of benign lesions will become a trend.

Peripheral blood SII, PLR, and NLR are considered as a novel inflammatory marker [12]. Studies have shown that NLR is elevated in patients with Hashimoto's thyroiditis and in diabetic patients with poor glycemic control [13, 14]. PLR has been reported to be associated with liver fibrosis in chronic hepatitis B [15]. Recently, studies have shown that inflammation plays an important role in the occurrence, development, and prognosis of cancer [16–18]. Also, Aktas et al. [19] and Cheong et al. [20] found that the inflammatory burden is increased in patients with malignant thyroid nodules. On this basis, this study was designed to further explore and validate the peripheral blood platelet count, NLR, PLR, SII, and other new inflammatory markers to predict the benignancy and malignancy of TI-RADS grade 3 thyroid nodules.

## 2. Materials and Methods

### 2.1. Patients and Methods

This study retrospectively analysed 514 patients who underwent surgical treatment or fine-needle aspiration for TI-RADS grade 3 due to various factors from January 2017 to October 2019 in the General Surgery Department or endocrine clinic of the First Affiliated Hospital of Nanchang University. All patients included in this study were older than 18 years and had thyroid function parameters within the normal ranges. The classification criteria developed by Kwak et al. [21] in 2011 were used to evaluate the internal composition, echo, margins, calcification, and shape of the nodules on ultrasound imaging. The exclusion criteria were as follows: (1) history of head and neck radiation; (2) recent infectious diseases; (3) history of thyroid surgery and other thyroid diseases; (4) blood system diseases, other tumours, coronary heart diseases, autoimmune diseases, and liver and kidney dysfunctions; The patients' basic information, laboratory test results, and pathological results were extracted from the medical records. Data on platelet count, neutrophil count, lymphocyte count, and thyroid function (reference range of normal values are as follows: FT3: 2.0–4.4 pg/mL; FT4: 0.93–1.70 ng/dL; TSH: 0.27–4.2 uIU/mL) were all obtained from blood tests in the morning on the second day of admission. The NLR, PLR, and SII were calculated as follows: NLR = neutrophil count ÷ lymphocyte count; PLR = platelet count ÷lymphocyte count; SII = neutrophil count × platelet count ÷ lymphocyte count. The research protocol was approved by the Ethics Committee of the First Affiliated Hospital of Nanchang University.

### 2.2. Statistical Methods

All data analyses were performed using Statistical Package for the Social Sciences, version 25.0, and *P* < 0.05 were used to denote statistical significance. The Kolmogorov–Smirnov was used to analyse the normal distribution of the data between groups. All measurement data in this study were not normally distributed. Therefore, the measurement data were represented as the median (25^th^–75^th^ percentiles). The distributions of continuous variables between two independent groups were compared using the Mann–Whitney *U* test. The chi-square test was used for comparing count data between two groups. Univariate and stepwise multivariate logistic regression analyses were performed to evaluate and determine the influencing factors of malignant thyroid nodules. The receiver operating characteristic (ROC) curve was used to analyse the effects and cutoff values of SII and age in predicting malignant thyroid nodules and calculate their sensitivity and specificity, positive predictive value (PPV), and negative predictive value (NPV).

## 3. Results

### 3.1. Patient Clinical Characteristics

In this study, 514 patients were enrolled, including 102 males and 412 females. Among these patients, 140 had malignant thyroid nodules (26 males and 114 females). Among them, 31 had lymph node metastasis. The median age of the patients in the malignant nodule group was 45.00 years. The benign nodule group comprised 374 patients, including 76 males and 298 females, with a median age of 50.00 years. No statistically significant difference in gender was observed between the malignant and benign nodule groups; however, the patients' ages in the malignant nodule group were significantly younger than those in the benign nodule group (*P* < 0.0001). No statistically significant differences in smoking, hypertension, or diabetes were observed between the malignant and benign nodule groups (*P* > 0.05) (Table 1).

Between the benign and malignant nodule groups, certain differences in various indicators were observed. In this study, no significant difference in thyroid function levels (FT3, FT4, and TSH), erythrocyte count, haemoglobin, and lymphocyte count were observed between the two groups (*P* > 0.05); however, the malignant nodule group had significantly higher leukocyte count, neutrophil count, platelet count, NLR, PLR, and SII than the benign nodule group (*P* values were 0.006, <0.001, 0.017, <0.001, 0.031, and <0.001, respectively). In this study, the number and size of thyroid nodules were also analysed, and it was found that the diameter of malignant nodules was significantly smaller than that of benign nodules, while the difference in the number of thyroid nodules between them was not statistically significant (Table 1).

### 3.2. Analysis of Influencing Factors for Malignant Thyroid Nodules

Many factors can affect the benignancy and malignancy of thyroid nodules. In this study, benign and malignant thyroid nodules were used as dependent variables, and age, gender, TSH, SII, leukocyte count, platelet count, neutrophil count, lymphocyte count, PLR, NLR, and the number and size of thyroid nodules were used as independent variables to perform univariate logistic regression analysis. The results showed that age, leukocyte count, platelet count, neutrophil count, PLR, NLR, and SII can affect the benignancy and malignancy of thyroid nodules (*P* values were <0.001, 0.006, 0.035, <0.001, 0.041, <0.001, and <0.001, respectively) (Table 2). To exclude the influence of confounding factors, we performed a stepwise multivariate logistic regression analysis of these possible influencing factors and found that SII (odds ratio (OR) = 1.006; 95% confidence interval (CI) = 1.003–1.008; *P* < 0.001), PLR (odds ratio (OR) = 0.981; 95% confidence interval (CI) = 0.981–0.992; *P* < 0.05), leukocyte count(odds ratio (OR) = 0.654; 95% confidence interval (CI) = 0.466–0.892; *P* < 0.05), and age (OR = 0.969; 95% CI = 0.954–0.985; *P* < 0.001) are independent influencing factors for malignant thyroid nodules (Table 2).

### 3.3. The Best Cutoff Value for Each Independent Influencing Factor to Predict Benign and Malignant Thyroid Nodules

**Figure 1:**
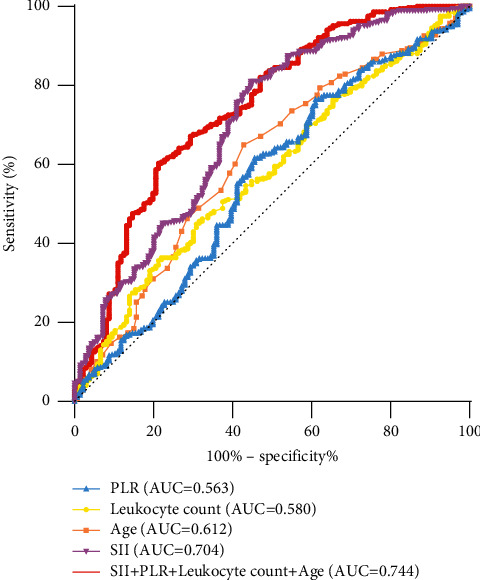
ROC curve of each independent factors.

The ROC curve analysis of independent risk factors affecting the nature of thyroid nodules (Figure 1) showed that the area under the ROC curve (AUC) of age, SII, PLR, and leukocyte count were 0.612,0.704, 0.563, and 0.580, respectively (Table 3). According to the Youden index, the critical values for age, SII, PLR, and leukocyte count to predict benign and malignant thyroid nodules were 46.5, 545.63 × 10^9^/L, 138.63, and 4.77 × 10^9/L, respectively (Table 3). Then, we performed ROC curve analysis on the combination of independent risk factors for thyroid malignant nodules and found that the AUC after the combination of SII, age, PLR, and leukocyte count was the largest (0.744) (Table 3 and Figure 1).

## 4. Discussion

Our results showed that the peripheral blood leukocyte count, neutrophil count, platelet count, PLR, NLR, and SII in the malignant nodule group were significantly higher than those in the benign nodule group, and SII, PLR, leukocyte count, and age were independent risk factors for malignant thyroid nodules. Therefore, for younger patients with TI-RADS grade 3 thyroid nodules with high SII, PLR, and leukocyte count, we recommend performing further diagnosis and treatment.

Various factors can affect the benignancy and malignancy of thyroid nodules. Studies have shown that women and older age are risk factors for malignant thyroid nodules [22], whereas the studies by Rago et al. [23] and Tuttle et al. [24] have shown that male patients, younger ones, and those with high TSH levels are more susceptible to have malignant thyroid nodules. Consistent with the results found by Rago et al. [23] and Tuttle et al. [24], this study also showed that younger age is a risk factor for malignant thyroid nodules; however, this study did not find significant differences in gender and TSH levels between the benign and malignant nodule groups.

The relationship between inflammation and cancer is a current research hotspot. An increasing number of studies have proven that inflammation and cancer are closely related. Inflammation is a promoter of tumour occurrence and development; moreover, malignant tumours themselves can stimulate the production of inflammatory markers [25]. Platelet and neutrophil counts have been reported to be elevated in patients with malignant tumours, which are also predictors of poor prognosis [26]. In this study, the levels of both were higher in patients with malignant thyroid nodules than those in patients with benign thyroid nodules. Current studies have highlighted that SII, NLR and PLR are new markers of peripheral blood inflammation. An increase in the level of NLR represents not only a decline in the body's immunity but also the enhancement of the tumour's ability to resist the body's immunity [12]. Studies have shown that NLR is higher in patients with malignant tumours than that in patients with benign tumours [27]. The study by Azab et al. [28] has shown that an increase in NLR is an independent predictor of high mortality in patients with breast cancer. For thyroid tumours, Kocer et al. [29] analysed 232 patients with thyroid nodules and showed that NLR levels were elevated in patients with papillary thyroid carcinoma. Liu et al. [30] have found that high NLR levels are associated with recurrence and poor prognosis in thyroid cancer. However, Haider et al. [31] showed that there was no significant difference in NLR between patients with benign and malignant thyroid nodules. In this study, although the level of NLR in the malignant nodule group was significantly higher than that in the benign nodule group, it was not an independent influencing factor for benign and malignant thyroid nodules.

In a retrospective study involving 354 patients with colon cancer, Hu et al. [32] have showed that PLR was significantly higher in patients with malignant colon cancer. The study by Tazeen et al. [33] has also shown that a high preoperative PLR level is an independent predictor of poor prognosis of oral squamous cell carcinoma. The study by Jan et al. [34] has shown that a high PLR level is associated with poor prognosis of urothelial cancer. Tel et al.'s [35]study showed that the PLR in the malignant thyroid nodule group was significantly higher than that in the benign nodule group, which was consistent with our findings. Moreover, our study also shows that PLR is an independent factor influencing malignant thyroid nodules and the optimal cutoff value was 138.63.

Compared with neutrophils, NLR, PLR, and other inflammatory indicators, SII may reflect the inflammatory state of the entire body more comprehensively. Moreover, studies have highlighted that SII is significantly increased in patients with malignant tumours [12]. Presently, no study has focused on the relationship between SII and thyroid nodules. In this study, SII in patients with malignant thyroid nodules was significantly higher than that in patients with benign thyroid nodules and was found to be an independent influencing factor for malignant thyroid nodules. The cutoff SII value for predicting malignant thyroid nodules was 545.63 × 10^9^/L. However, its best cutoff value should be further verified by conducting studies with larger sample sizes. TI-RADS grade 3 thyroid nodules do not need to be overdiagnosed and treated and only need regular monitoring; however, it is inevitable that a small part of thyroid cancer is missed [11]. The measurement of peripheral blood cells is an affordable and convenient method. Clinically, we suggest that peripheral blood cell determination should be performed routinely in patients with thyroid nodules. The patients with TI-RADS grade 3 thyroid nodules with higher SII, PLR, leukocyte count, and younger age should undergo fine-needle aspiration, contrast-enhanced ultrasonography, or other diagnostic modalities to confirm the nature of the nodule, rather than going directly into regular monitoring stage.

This study has several limitations to consider. First, this is a retrospective study, which may have a selection bias. Second, the sample size of this study is not large, which may cause statistical errors. Third, patients with malignant nodules have not been followed up, and the prognosis of these patients cannot be further investigated.

## 5. Conclusion

In conclusion, this study showed that the peripheral blood leukocyte count, neutrophil count, platelet count, PLR, SII, and other inflammatory markers in the malignant thyroid nodule group were significantly higher than those in the benign nodule group, and SII, PLR, leukocyte count, and age were independent risk factors for malignant thyroid nodules. Peripheral blood SII, PLR, leukocyte count, and age can jointly predict the benignancy and malignancy of TI-RADS grade 3 thyroid nodules. This simple test can further guide the diagnosis and treatment of this type of thyroid nodule. However, the exact relationship between inflammation and benign and malignant thyroid nodules should be further verified by large-scale studies.

## Figures and Tables

**Table 1:** Clinical characteristics of benign and malignant thyroid nodules.

	Malignant nodules group (*n* = 140)	Benign nodule group (*n* = 374)	*P* vaule
Gender (male/female)	26/114	76/298	0.658
Age (years)	45.00 (36.00–54.75)	50.00 (43.00–60.00)	<0.001^*∗*^
Hypertension (yes/no)	20/120	51/324	0.841
Diabetes (yes/no)	13/127	22/352	0.173
Smoking (yes/no)	9/131	30/344	0.544
Solitary nodule (yes/no)	48/92	129/245	0.965
FT3 (pg/ml)	3.20 (2.96–3.51)	3.19 (2.90–3.45)	0.223
FT4 (ng/dl)	1.30 (1.12–1.40)	1.29 (1.17–1.42)	0.780
TSH (uIU/ml)	1.62 (1.05–2.46)	1.18 (1.77–2.71)	0.187
Leukocyte count (×10^9^/L)	5.65 (4.85–6.80)	5.36 (4.45–6.32)	0.006^*∗*^
Erythrocyte count (×10^9^/L)	4.36 (4.18–4.66)	4.36 (4.05–4.63)	0.120
Platelet count (×10^9^/L)	240.50 (204.25–279.50)	228.50 (191.25–265.00)	0.017^*∗*^
Hemoglobin (g/L)	131.00 (124.00–139.00)	129.00 (120.00–137.00)	0.071
Neutrophil count (×10^9^/L)	3.40 (2.75–4.33)	2.99 (2.35–3.76)	<0.001^*∗*^
Lymphocyte count (×10^9^/L)	1.72 (1.41–2.06)	1.73 (1.46–2.12)	0.464
NLR	2.02 (1.34–2.73)	1.66 (1.30–2.10)	<0.001^*∗*^
PLR	141.02 (105.43–182.20)	127.11 (104.32–158.89)	0.031^*∗*^
SII (×10^9^/L)	457.68 (300.37–682.70)	378.46 (275.02–494.03)	<0.001^*∗*^
Diameter of nodule (cm)	2.00 (1.00–3.60)	2.50 (1.70–3.40)	0.019^*∗*^

^*∗*^ *P* < 0.05.

**Table 2:** Univariate and multivariate logistic regression models in malignant influencing factors of thyroid nodules.

	Univariate analysis		Multivariate analysis	
	OR (95% CI)	*P* value	OR (95% CI)	*P* value
Gender	0.894 (0.545–1.467)	0.658	NA	NA
Age	0.972 (0.958–0.987)	<0.001^*∗*^	0.969 (0.954–0.985)	<0.001^*∗*^
Solitary nodule	0.991 (0.658–1.492)	0.965	NA	NA
TSH	0.994 (0.844–1.056)	0.316	NA	NA
Leukocyte count	1.222 (1.060–1.409)	0.006^*∗*^	0.654 (0.466–0.892)	0.008^*∗*^
Platelet count	1.003 (1.000–1.007)	0.035^*∗*^	NA	NA
Neutrophil count	1.482 (1.247–1.763)	<0.001^*∗*^	NA	NA
Lymphocyte count	0.882 (0.595–1.306)	0.529	NA	NA
NLR	1.640 (1.315–2.045)	<0.001^*∗*^	NA	NA
PLR	1.004 (1.000–1.008)	0.041^*∗*^	0.981 (0.969–0.992)	0.001^*∗*^
SII	1.003 (1.002–1.003)	<0.001^*∗*^	1.006 (1.003–1.008)	<0.001^*∗*^
Diameter of nodule	0.923 (0.788–1.018)	0.320	NA	NA

**Table 3:** ROC curve analysis of each independent influencing factor.

	AUC	*P* Value	95% CI	Cutoff value	Sensitivity (%)	Specificity (%)	PPV (%)	NPV (%)
SII	0.704	<0.001^*∗*^	0.651–0.757	545.63 × 10^9^/L	55.4	88.1	62.93	84.07
Age	0.612	<0.001^*∗*^	0.557–0.667	46.5 years	65.0	57.1	36.19	81.34
PLR	0.563	0.031^*∗*^	0.504–0.621	138.63	54.4	61.6	32.40	77.10
Leukocyte count	0.580	0.006^*∗*^	0.525–0.635	4.77 × 10^9^/L	78.7	35.9	31.49	81.83
SII + PLR + leukocyte count + age	0.744	<0.001^*∗*^	0.693–0.794	—	78.7	60.3	42.60	88.32

## Data Availability

The data used to support the findings of this study are available from the corresponding author upon request.
